# Compatibility of Cats With Children in the Family

**DOI:** 10.3389/fvets.2018.00278

**Published:** 2018-11-19

**Authors:** Lynette A. Hart, Benjamin L. Hart, Abigail P. Thigpen, Neil H. Willits, Leslie A. Lyons, Stefanie Hundenski

**Affiliations:** ^1^Department of Population Health and Reproduction, School of Veterinary Medicine, University of California, Davis, Davis, CA, United States; ^2^Department of Anatomy, Physiology and Cell Biology, School of Veterinary Medicine, University of California, Davis, Davis, CA, United States; ^3^Department of Statistics, University of California, Davis, Davis, CA, United States; ^4^Department of Veterinary Medicine and Surgery, College of Veterinary Medicine, University of Missouri, Columbia, MO, United States

**Keywords:** cat aggressive behavior, cat affectionate behavior, cat fearfulness, cats and children, cultural differences, human-animal interaction, anthrozoology

## Abstract

Although studies involving pet dogs and cats, and human adults and children, have been reported, the specific interactions between cats and children have not. This study sought information from parents about the cat's role in families that have at least one child 3–12 years of age and at least one cat. Demographic data on cat source, breed, gender/neuter status, was sought as well as information on adults and children in the families and on affectionate, aggressive, fearful, and playful responses of the cats to children. A convenience sample was recruited via listservs for pet owners and parents. Using a pilot tested web survey, descriptive statistics were based on 865 respondents. Multi-variate statistical analyses were conducted on data from 665 respondents with complete responses for all items, including respondents' locations and whether cats were adopted as kittens. Multi-variate analyses included consideration of demographic data, geographic region of respondents, behavioral characteristics of the cats, and responses of the children to the cats. From descriptive statistics, cats' affection was more typical with adults than young children. Neuter status or gender was unrelated to cats' aggression or affection. Being the family's only cat was associated with heightened aggression and reduced affection. Younger cats were more likely to be affectionate. Multivariate analysis revealed three primary factors accounting for children's compatibility with the specified cat: positive interactions of the cat, aggression/fearfulness of cat, and the cat's playfulness and children's reaction to the cats. Positive child-cat relationships were more typical with two or more adults and multiple cats in the home. Old cats were the least satisfactory. A breeder or shelter was a better source than as a feral, from a newspaper ad, or another source. European respondents rated their cats' interactions with children more favorably than in U.S./Canada. This difference may reflect the European adoptions more frequently being of kittens, often purebred, assuring more early handling within the family. A noteworthy finding was that all family participants, humans, and pets alike, affect the cat-child relationship, and these results reveal that many variables can play a role in achieving a desirable relationship for a cat and child.

## Introduction

Many studies have documented the contributions of pets to children's emotional and physical development (1). The value of cats as pets has been extensively studied over decades, focusing on their interactions with adults (2) and documenting contributions to human health (3). With regard to children's pets, studies often have examined the development of empathy among children who nurture pets. Yet, as revealed in reviews, most of these studies do not treat dogs and cats separately, but rather lump dogs and cats together as companion animals or pets (4, 5), despite evidence that dogs and cats clearly differ (6). Often, dogs are emphasized as a major focus, perhaps because they frequently emerge as the preferred pet, as shown in an early study (7), and in examples from U.S. (8) and Holland (9). Thus, despite many studies exploring children's interests and engagement with pets, little specific attention has been directed to understanding details of cats' behavioral responses to children and children's relationships with cats.

In assessing children's interest in pets, Brucke (7) evaluated the essays of 7–16 year old children about pets and noted that more children preferred dogs than cats and interest in them increased as the children got older. In another study, children 3–5 years of age, in choosing between paired photographs, showed a preference for more infantile over adult cats; similar differences were not found when they were asked to choose between infantile and adult dog photographs (10).

Many studies by Levinson (11) expanded on the importance of pets for children in filling several roles, including as companions and confidants. Focusing on adolescents' loneliness and companion animals, Black (12) presented a conceptual model of the contributions of companion animal attachment that included constituents of caregiving, offering a secure base and safe haven, and proximity seeking and separation anxiety in the absence of the animal.

Studies reported that virtually all children were found to want a pet (13), and children lacking pets often desired one and sought out contact with their neighbors' pets (14). In interviews of Pennsylvania latchkey children (8) with a median of 8 years of age, dogs were the primary pet and the most frequently owned. However, for the children without pets, cats were wished for most, with dogs second.

In evaluating children's drawings of themselves and their other family members, a study found that children who owned pets placed the drawing of themselves significantly closer to their drawings of their pets than to other members of their family (15). The closeness at which they placed themselves to cats vs. dogs did not differ, suggesting that the children experienced the supportive characteristics of cats and dogs similarly and that closeness was based on animals' general characteristics. The same authors (16) also found that 3–7 year-old children's experiences at a petting zoo where they could easily see and touch the animals was associated with forming favorable attitudes toward animals more than when they visited large wildlife exhibits. When they studied toddlers responding to live and toy animals, even very young infants strongly preferred live pets to mechanical animals, and spent significantly more time observing and interacting with the live animals (17). Children 12–18 months old used the animal's species name, and by 24–30 months, the children called the animal by its given name. The 12–30 month-old children preferred dogs to cats, presumably because the dogs were interactive and more likely to approach the children, whereas the cats often walked away, thus limiting reciprocal interaction. As noted by these authors in their studies of parent-child relationships, this reciprocal interaction is a characteristic of attachment (18, 19), and the same may be true in child-pet relationships. Turn-taking of this sort forms a basis for communication, one in which conversational interchange becomes possible (20). Recent studies of human-cat relationships have emphasized that both the cat and the human affect and contribute to the relationship and bond involved (21, 22).

Triebenbacher (23) described the importance of transitional objects to which children become attached as they begin separating from their parents; these soothe and calm the children. While blankets and cuddly toys commonly serve as transitional objects, pets also fill this role. Preschool children articulated their specific emotions characteristic of special relationships, especially that these relationships were reciprocal; they understood that the best way to show love is through affection. Children in grades 2 through 5 most commonly said if their pets could talk they would say, “I love you.” (23). As special friends and important family members, pets also provide affection, social interactions, and emotional support.

When considering the most important relationships in their lives, 42% of 9–12 year olds in one study chose a pet, more often or the same as grand parents, aunts, uncles, friends or teachers (24). Similarly, in an earlier study, 7 and 10 year-old children taking a neighborhood walk listed a pet among their special friends (25).

Interest in dogs and cats can change with age (26). Parents reported that kindergarteners were more involved with and more interested in dogs than cats, while the reverse was true of second-graders. Among fifth-graders, there was no difference reported in involvement or interest between dogs and cats. In kindergarten, second- and fifth-grades, questionnaire responses were collected from one parent of each child. Older children and those whose mothers were employed were more attached to their pets. Moreover, ideas about pets and their care appear to generalize beyond the specific type of pet owned. Children who owned dogs but not cats were reported to be just as knowledgeable about cats and their care as were cat-owners. And similarly, cat-owners showed as many ideas about dogs and their care as did dog owners. In a study by Daly and Morton (27), higher empathy was found for children owning both dogs and cats as compared with children owning neither a dog nor a cat, or only one type of pet. Children that were highly attached to their pets were more empathic than those who were less attached.

Various approaches have categorized types of behaviors of cats. In a study of domestic cat personality, adjectives included *amiability*, which was strongly positively correlated with owner satisfaction, attachment, and bond quality. *Amiability* included descriptors such as cooperative, warm, peaceful, charming, and faithful (28). *Demandingness* included: persistent, demanding, needy, persevering, and loud. *Dominance* included: proud, domineering, serious, independent, and territorial. *Nervousness* included: nervous, timid, apprehensive, and cautious. Neutered (spayed) females scored higher on demandingness than intact males. *Amiability* increased with cat age, decreased with owner age, and increased with the number of cats in the home. In a somewhat similar study, personality attributes of cats [(29), p. 157] were used to measure sociability of cats toward humans. In assessing cats for their behavioral tendencies prior to adoption to evaluate systems of seeking, fear, and rage, variables included *sociability, boldness, gregariousness, frustration reactivity, and fearfulness*.

Yet another approach assessing personality qualities of cats generated factors using a principal components analysis based on observer ratings and behavior codings (21). Four factors involved in social interactions included *active, anxious, sociable*, and *rough*. Subtle behavioral indicators of fear vs. engagement were identified in a recent study of cat behavior, showing that a left gaze and head turn reflects fear, whereas a right gaze and head turn reveals engagement (30).

Early experience with being handled during a sensitive period is known to increase friendly and affectionate behavior in cats (2, 31). Kittens that have been handled often more rapidly approach a familiar person, a stranger or a novel object as compared with unhandled kittens (32). When a UK shelter offered enhanced socialization to kittens between 2 and 9 weeks of age, owners interviewed when the cats were ~1 year of age reported they felt more emotionally supported by their cats; fewer of these cats were fearful with humans, as compared with cats that had received standard handling (33).

In Australia, a study of 488 people with cats found that almost all cats were neutered; only 3% of owners reported having an intact male cat, and 2% reported owning an intact female cat (34).

In New Zealand, a study of children 8–12 years old found that cats were the most frequently owned pet; they were owned by 71% of families (35). A majority of children in the cat-owning families were said to be “owner” of the cat. This was particularly true in families with only one or two children The child wanted the cat and it was often acquired to teach responsibility. In a UK study, those in semi-urban and rural householders more often reported cat ownership, as did returns from female respondents (36). In a longitudinal study of UK parents with children up to 10 years of age, cats were the most commonly owned pet, and cats were most common in families with female children (37). In another UK study, with children of all ages, cats were found in the highest number of households. In a Norway study of children and adolescents aged 9 to 15, a majority of rural participants had a cat (38).

A study of UK adolescents who had only one pet reported that 57% of respondents had a dog and 23% a cat (39). For those with multiple pets, a dog and cat was the most frequent combination. Adolescents that lived in single-parent families or stepfamilies more often reported having pet cats, when they were compared with adolescents living with two parents. Also, cat ownership was more often reported by adolescents with siblings than by those who had no siblings. Adolescents reporting a median or higher level of family affluence less often reported having a cat than those reporting a low level of family affluence.

In a study of cats in Italy, the owner's gender influenced the cat's time spent with the owner: cats spent more time with women than men (40). The composition of the family influenced the cat's behavior toward the owner and the time spent with the owner. The more sociable cats lived in small families having no children. The cats that lived with other cats had a higher quality of life, based on care, behavior, and a physical examination, than those cats living alone. Cats generally are not thought to be highly social, but it seems that living with other cats may improve a cat's quality of life. In this study, cat owners who adopted their kittens between 7 and 10 weeks of age were more attached to their cats later on than the owners who had adopted older cats. This young age of adoption appears to be an important time to let the cat socialize with humans and with animals of other species in the household.

The number of human adults in the family seems to play a role in attachment to the cat, as suggested in a study in Switzerland, where families with fewer adults reported higher attachment to the cat than in families with more adults (41). The size of the household was negatively correlated with two attachment scales.

In Japan, families with dogs often considered their pets to be family members, but families with cats less often held this view (42). Compared with dog owners, cat owners scored their pets lower on emotion and intellect. Those cat owners who considered their cats to be family members were more likely to attribute compassion to their cats when compared with owners who regarded their pets as not being family members.

For this study, we hypothesized that a well-mannered cat that can be held by a child could be a valuable companion. While cats typically rest much of the day, at times, cats could be significant companions for children, being a source of calming comfort. Despite abundant evidence that pets matter to children, most reports on children and pets somewhat lump together dogs and cats rather than specifically examining the interactions of cats and children, or the behaviors or preferences that children may have for cats.

The study sought to characterize the interactions of cats with children compared with their interactions with adults in families responding to a general web survey.

Cat breeds differ in their behavioral tendencies, such as their affection and aggressive behaviors toward family members (43); one would expect the breed of cat to be one aspect affecting which cats would provide affection and comfort to a child. For example, a comprehensive telephone-based set of interviews with 80 feline veterinary practitioners covering 15 of the most common cat breeds, found that the Ragdoll is the most affectionate, socially outgoing and least aggressive breed (43). The same survey found that male cats were rated as more affectionate than female cats.

In this study we gathered data from the general public, by means of a web-based survey, to determine factors that would predict, or correlate with, the characteristic of cats being affectionate and non-aggressive with children. Overall, the study focused on cats' behaviors with children to characterize and determine behavioral correlates and attributes of positive relationships with cats. Of particular interest also was the extent to which children in the family valued the relationship with the specified cat.

## Methods

### Web-based survey of families with a child, 3–12 years of age, and with a cat

A web-based survey was designed in SurveyMonkey to gain information about cats' characteristics that qualify them as desirable companions for young children. The 25 item survey was directed toward families, most presumably with typically developing children, and launched to appropriate listservs, publicizing and disseminating the link to feline and parent groups and other cat-interested groups. It was required that participants have a child within 3–12 years of age and a cat that was at least 1 year of age. Families with multiple cats were instructed to answer questions pertaining to the cat most interactive with the child/children in the family, to characterize behaviors in the more interactive cat/child relationships, rather than an average cat/child relationship that would have included the more outdoor and fearful cats. The survey was designed to require about 15 min to complete. Numerous published reports of behavioral studies have used similar web-based surveys [e.g., (44)]; such surveys have been found comparable in validity to more traditional survey methods (45). This survey was open for responding October 2010 through January 2012.

Among the 1,000 respondents allowed to complete this survey prior to ending data collection, 865 met the inclusion criteria: having at least one child 3–12 years old; having in the household at least one cat that was at least 1 year of age; and completing all of the 25 questions of the survey. The socio-demographic information gathered included: numbers of adults in the family; ages of children; and information on the numbers of cats and dogs in the household, as well as the age, breed and source of the cat specified as interacting the most with the children in the family. Parents provided specific behavioral ratings for the cat and children's responses to the cat on a five-point scale. For example, the cat's affectionate interactions were categorized as: very affectionate; quite affectionate; moderately affectionate; relatively non-affectionate; and non-affectionate. The cat's aggressive interactions were categorized as: very aggressive; quite aggressive; moderately aggressive; relatively non-aggressive; and non-aggressive. Parents also provided ratings of the children's level of interest in the cat.

### Institutional review approval board

The University of California, Davis, Institutional Review Board approved Protocols #201018447-1 and #284059-2.

### Statistical analyses

#### Descriptive statistics

Descriptive statistics were used to analyze data from 865 participants, including medians and results of chi-square or Fisher exact tests for significance. Further multivariate analyses from 665 participants included geographic location of respondents based on IP addresses, and whether adopted as kittens when specified in responses. Inclusion criteria for the multivariate analyses required specific answers: some participants' responses were excluded due to responding with the item, “other.”

#### Multivariate statistics

For the survey data, thirteen responses were identified as reflecting the quality of interactions between the family children and the focal cat. These included: affection, aggression, friendly behavior, playfulness, and fearfulness toward various age groups of children and adults, as well as the child's reaction to the cat. A principal component analysis (PCA) was run on these variables, and the first three principal components, which explained 62% of the variability in the responses, were used in additional analyses. In this analysis, only subjects that answered all 13 questions were used, reducing the sample size to 665 responses. The factor loadings for the first factor, named *cat's positive interactions*, were all positive except for two variables reflecting fearfulness, which were negative. The second factor contrasted two negative behaviors (aggression and fearfulness) against more positive behaviors, and the third paired the child's reaction to the cat and positive behaviors like playfulness against the cat's aggression, particularly toward children. The eigenvectors for the principal components, and their values for each of the survey responses, are available in Excel files. Each of the first three factors were used as dependent variables in several one-way ANOVA models, looking for systematic differences with respect to a series of demographic variables, indicating the global region from which the survey response came, the composition of the family in which the cat lived, the source of the cat, the cat's current age, whether adopted as a kitten, and gender and neuter status of the cat. For this analysis, global regions were consolidated as: U.S/Canada, Europe, other. Cat breeds were consolidated as: mixed (including domestic shorthair and domestic longhair) and purebred. Factors that were statistically significant for one or more of the first three PCAs were presented in a biplot of the factor loadings and group differences. Those factors were also used as dependent variables in a conditional inference tree analyses (a form of CART) that used a broader array of explanatory variables. All analyses were run using SAS, version 9.4, except for the inference trees, which were run using R statistical software and the ctree command.

#### Data availability

In compliance with journal policy, datasets used for statistical analyses, including the PCA factor loadings, are publicly available at figshare.com: http://dx.doi.org/10.6084/m9.figshare.7007993.

## Results

### Descriptive statistics

#### Family and pet demographics

The survey with 865 families meeting inclusion criteria revealed that most of the children resided in households with at least two adults available; 12 percent of families included only one adult.

Regarding the age-ranges of children, 28 percent of households had teenagers 13–19 years of age, 36 percent had children 9–12 years, 31 percent had children 6–8 years, and 40 percent had children 3–5 years. Multi-pet households were a majority of respondents, with 63 percent having multiple cats over 1 year of age. Forty-seven percent had at least one dog, of which 48 percent had multiple dogs.

The survey focused on the cat that interacted with the children the most. Almost one-third of these cats, 31 percent, were 3 years of age or less; 59 percent of cats were 6 years old or less. Male neutered cats comprised 49 percent and female spayed cats 46 percent of the specified cats, with 2 percent being intact males and 4 percent being intact females. With regard to breed, 57 percent were domestic shorthair, 11 percent domestic longhair, and 6 percent Maine Coon.

#### Cat's affectionate, fearful, or aggressive behavior as related to the cat's age and gender and children's ages

While 32 percent of the designated cats were very affectionate toward adults and 27 percent toward 6–12 year olds, just 14 percent were very affectionate toward 3–5 year olds (statistical tests: 3 groups, or 2 pairs, all *p*s < 0.0001). With regard to the age of the cat, the 78 cats that were very affectionate toward 3–5 year olds were younger, with 58 of 78 (74%) being 6 years of age or less, as compared with 255 of 469 (54%), fewer, of the remaining less affectionate cats being 6 years of age or less (*p* < 0.001). The cats that were very affectionate consisted of 51 percent neutered males and 42 percent spayed females, with no intact males and 6 percent intact females.

Among cats rated as very affectionate to adults and/or children (*n* = 360), 239 (66.4%) were 6 years of age or less and 121 (33.6%) were 6 years or older. Among the remaining 505 cats that were not rated as very affectionate, 270 (53.5%) were 6 years of age or less and 234 (46.3%) were older than 6 years. Very affectionate cats were significantly younger than the other cats (*p* < 0.001).

Among 360 cats rated as very affectionate with children and/or adults, 278 (77.2%) were described as very affectionate to adults, 171 (47.5%) as very affectionate to children 6–12 years of age, and only 78 (21.7%) to children 3–5 years of age (adults vs. children, children vs. children: all *p*s < 0.0001). Very affectionate cats were more likely to express affection toward adults than toward children; they were least likely to express affection toward the youngest group of children.

Ratings of cats being very fearful were infrequent: among the 78 cats very affectionate with 3–5 year old children, only 2 (3%) were fearful of visiting children. Among the cats rated as anything less than very affectionate with 3–5 year olds, 54 of 469 (11.5%) were described as “very fearful, runs away and stays hidden” with visiting children (*p* < 0.0001). Further, among the 87 cats described as “definitely does not like being held or carried around” by children ages 3–6, 25 (28.7%) were also described as very fearful.

While 56 of 78 (71.8%) very affectionate cats came from multi-cat households, only 284 of 469 (60.6%) less affectionate cats came from multi-cat households. Interestingly, cats that were very affectionate toward 3–5 year olds were not always affectionate toward adults, as illustrated by our finding that among 78 cats very affectionate toward 3–5 year olds, only 50 cats (64%) were also very affectionate toward adults.

The median age range of cats rated as at least moderately aggressive to adults, children, or other cats (*n* = 63) was 7–10 years. Of these aggressive cats, 27 (42.9%) were 6 years of age or less, and 35 cats (55.6%) were older. One respondent was too unsure of the cat's age to indicate a range. Among the remaining 802 cats not rated as aggressive, 482 (60.1%) were 6 years of age or less, and 320 (39.9%) were older. Comparative analyses reveal that these aggressive cats were significantly more likely to be older (*p* < 0.01).

Among these 63 aggressive cats that were at least moderately aggressive, 29 (46%) were spayed females, 2 (3.2%) were intact females, 31 (49.2%) were neutered males, and 1 (1.6%) was an intact male. Among the remaining 802 cats not rated as aggressive, 366 (45.6%) were spayed females, 33 (4.1%) were intact females, 389 (48.5%) were neutered males, and 14 (1.7%) were intact males.

Among 24 cats scored as at least quite aggressive to children in the home, 15 (62.5%) were the only cat in the home and 14 (58.3%) were in homes without dogs; among the other 775 cats, 280 (36.1%) were the only cat and 407 (52.5%) were in homes without dogs. These aggressive cats were significantly more likely to be the only cat in the home (*p* = 0.01). These data suggest that these 24 quite aggressive cats tended to be isolated from other cats, but not dogs.

#### Cat's affection to adults and children

At least moderate affection was shown by 706 of 865 (81.6%) cats in this study to adults, as shown in Table 1. A somewhat lesser percentage of cats, but still a majority, was similarly affectionate to children: 429 of 626 (68.5%) for 6–8 year olds and 297 of 547 (54.3%) for 3–5 year olds.

**Table 1:** Percentages of cats rated as moderately affectionate to children and adults: web survey of general public.

	**Cats at least moderately affectionate**		
**Web survey**	**Adults**	**Children 6-8 yrs**	**Children 3-5 yrs**
Public	706/865 (81.6%)	429/626 (68.5%)	297/547 (54.3%)

Considering these cats in families of the general public, neuter status, or gender was unrelated to the cats' aggression or affection. Being the family's only cat was associated with heightened aggression and reduced affection. Younger cats were more likely to be affectionate.

#### Cat's behavior affecting the child-cat relationship

In an open-ended item in the survey, parents had an opportunity to remark on the child's interaction with the designated cat that interacted the most with the child or children in the family. In these responses a vast majority of the children “liked to hold or sit with the designated cat about half the time,” or “usually loved to hold or pet the cat,” or were “crazy about holding, petting, snuggling and sleeping with the cat.” Among 792 parents, 638 (81%) rated their children as being at least moderately responsive to the cat half the time, indicating that most children sought and valued the relationship with the cat.

Among children living with the 63 cats rated as at least moderately aggressive cats, parents involved with 16 (25.4%) of these cats rated their children as crazy about the cat, and 6 (9.5%) parents described their children as feeling indifferent to the cat. One parent whose child was crazy for the cat wrote that, “the cat is not having it.” In comments, parents sometimes described a conflicted situation where the child: “would love to hold and cuddle the cat,” but “the cat views any interaction from the child as a potential threat,” “will leave if possible,” or “the cat hates it.”

As a contrast with the aggressive cats, of the 360 cats rated as very affectionate with children and/or adults, parents involved with 173 (48%) of these cats rated their children as crazy about the cat, and only 10 (3%) parents scored their children as indifferent. There was a highly significant level of compatibility of children who had affectionate rather than aggressive cats (*p* < 0.001). In the 6–12 year age group, parents involved with 190 cats in 488 (39%) households judged their children as crazy about the cat; this was a trend toward a higher percentage of children being crazy about the cat than reported by the parents involved with the 113 cats for younger children, 3–5 years of age, from 347 (33%) households.

Some cultural differences were evident, for 343 out of 776 (44.2%) cats in U.S./Canada were adopted from a shelter, compared to 11 out of 63 (17.5%) in Europe (*p* < 0.0001). Conversely, 196 out of 776 (25.3%) cats in the U.S./Canada were purebred, compared to 41 out of 63 (65.1%) in Europe (*p* < 0.0001). Additionally, only 172 out of 776 (22.2%) cats in the U.S./Canada were adopted as kittens, compared to 42 out of 63 (66.7%) in Europe (*p* < 0.0001).

To summarize, cats in the U.S./Canada were more likely than in Europe to come from shelters, and less likely to be purebred and adopted as kittens.

### Multivariate statistics

The Principal Components Analysis (PCA) revealed three primary factors; PCA Eigenvalues of the correlation matrix were: Prin 1, 4.72; Prin 2, 1.99; Prin 3, 1.29. Behaviors of primarily positive interactions of cats with children play a major role in the first behavioral factor, *cat's positive interactions* (e.g., from high to low), friendly to visiting kids, affection of the cat to kids 6–8 years old, to kids 3–5 years old, friendly to adults, affectionate to adults, affectionate to children; and with negative loadings for fearful with kids 3–5 years old and fearful with adults. These Prin 1 vectors then were associated in one-way ANOVA models with non-behavioral variables, such as the geographic region of the respondent, number and ages of children, the age and source of the cat, the age at adoption, the cat's breed and gender/neuter status. The second factor, *cat's fearfulness/aggression*, is dominated by; fearfulness with adults, aggression with kids 6–8 years old, aggression with adults, and fearfulness with kids 3-5 years old; this was associated in the ANOVA model with specific non-behavioral variables such as the ages of children, the sex status of the cat, and whether the cat was adopted as a kitten. The third factor, *cat's playfulness and child's positive reaction*, pertains to: the cat being playful with children 3–5 years old, being playful with adults, the children's positive reactions to the cat, and the cat's playfulness, with the cat's aggression with young children and adults having a substantial negative loading; this factor also was associated in the ANOVA model with the number and ages of children, and the cat's age.

**Figure 1:**
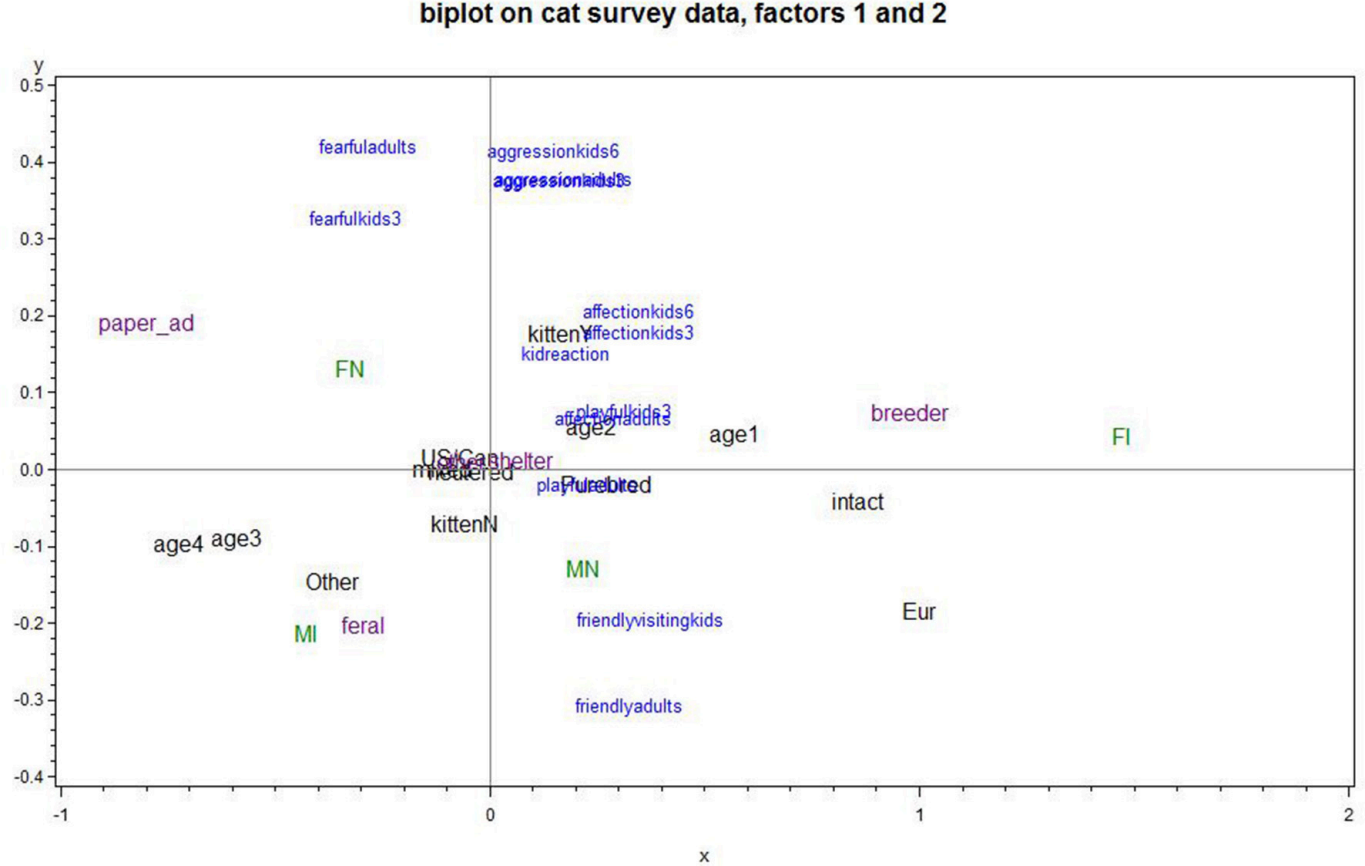
Biplot of Factor 1 cat's positive interactions and factor 2 cat's fearfulness/aggression (Factor 1 increases to the right; Factor 2 increases going up. Lower right quadrant is the optimal relationship; e.g., intact cats were better for positive interactions than neutered females; cats from a breeder provided more positive interactions than those from an ad; ferals scored low on fearfulness/aggression; cats in Europe scored better on these two factors than those scored in the U.S./Europe). Points plotted in blue represent the variables that were used in the PCA. Points plotted in green, purple or black place subgroups of responses on the graph, corresponding to neuter/gender status, cat source, and miscellaneous categorical predictors, respectively. Points that plot in the same general direction relative to the origin are positively associated, while ones that plot on opposite directions are negatively associated. The strength of an association is related to the distance from the origin, so the points closest to the origin exhibit negligible associations.

To illustrate these results more specifically, three biplot figures are presented; each is a biplot of two of the PCAs revealing group differences. The biplots highlight and focus on variables found to have significant differences and exclude those with only marginal differences. In plots the cat's age range is indicated by: age1, 1 up to 3 years old; age2, 3 up to 6 years old; age3, 6 up to 10 years old; age4, over 10 years. Figure 1 plots PCA Factors 1, *cat's positive interactions*, and 2, *cat's fearfulness/aggression*; the lower right quadrant is most favorable and the upper left is least favorable for a positive cat-child relationship. A cat living in Europe, or being intact or a neutered male, was associated with the cat's positive interactions and low fear/aggression. A cat being young, an intact female, or from a breeder was associated with the cat's positive interactions. Being adopted as a kitten was somewhat associated with lower fear/aggression. Conversely, a cat being a neutered female or acquired from a newspaper ad was associated with a cat's negative interactions and high fear/aggression. A cat being older, feral, or an intact male, is off to the left of the bi-plot and associated with a cat's negative interactions to children, but low fear/aggression.

**Figure 2:**
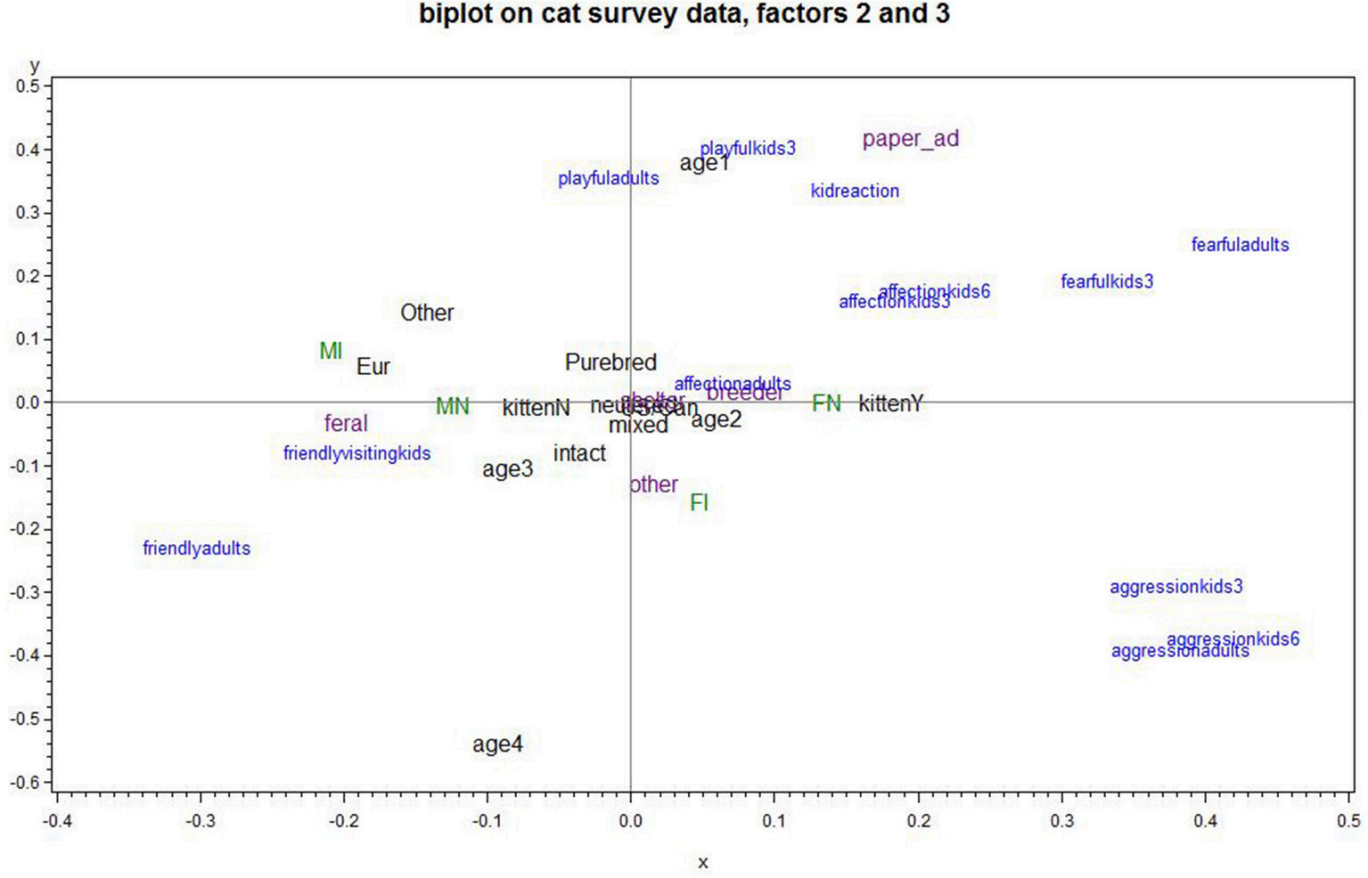
Biplot of Factor 2 cat's fearfulness/aggression and Factor 3 cat's playfulness and child's positive reaction (Factor 2 increases going to the right; Factor 3 increases going up). Upper left quadrant is optimal; e.g., male intact cats were better for a child's positive reactions than female intact or female neutered cats). Points plotted in blue represent the variables that were used in the PCA. Points plotted in green, purple or black place subgroups of responses on the graph, corresponding to neuter/gender status, cat source, and miscellaneous categorical predictors, respectively. Points that plot in the same general direction relative to the origin are positively associated, while ones that plot on opposite directions are negatively associated. The strength of an association is related to the distance from the origin, so the points closest to the origin exhibit negligible associations.

Figure 2 plots PCA Factors 2, *cat's fearfulness/aggression*, and 3, *cat's playfulness and child's positive reaction*; the upper left quadrant is most favorable and the lower right is least favorable for a positive cat-child relationship. A cat living in Europe or being an intact male scored low on fearfulness/aggression and somewhat positive for playfulness and the child's positive reaction. Cat's playfulness and positive reactions from children, but also with heightened fear/aggression, were associated with a cat's young age and being acquired through an ad. A cat being old was associated with somewhat low fear/aggression, as well as low playfulness and negative reactions from children. Kittens, neutered males, and feral cats showed increasingly low levels of fearfulness/aggression.

**Figure 3:**
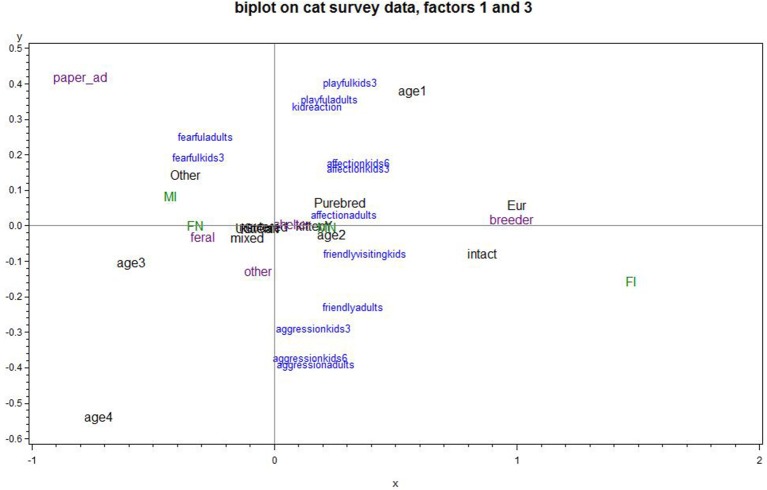
Biplot of Factor 1 cat's positive interactions and Factor 3 cat's playfulness and child's positive reaction (Factor 1 increases going to the right; Factor 3 increases going up). Upper right quadrant is optimal; e.g., an intact or female intact, a cat from a breeder, or a cat in Europe on average scored better than average for positive interactions; a cat from a newspaper ad scored worse. Adopting a young cat was associated with better scores on both factors than one adopted when older Points plotted in blue represent the variables that were used in the PCA. Points plotted in green, purple or black place subgroups of responses on the graph, corresponding to neuter/gender status, cat source, and miscellaneous categorical predictors, respectively. Points that plot in the same general direction relative to the origin are positively associated, while ones that plot on opposite directions are negatively associated. The strength of an association is related to the distance from the origin, so the points closest to the origin exhibit negligible associations.

Figure 3 plots PCA Factors 1, *cat's positive interactions*, and 3, *cat's playfulness and child's positive reaction*; the upper right quadrant is most favorable and the lower left is least favorable for a positive cat-child relationship. A cat being young was associated with positive interactions from the cat and reactions of the child, whereas the cat being old was associated with negative cat and child reactions. The cat living in Europe, being an intact female, or acquired from a breeder was associated with the cat's positive interactions. The cat being acquired from a paper ad was strongly associated with negative cat interactions but positive child reactions. The cat being an intact male, a neutered female, or feral was associated with the cat's negative interactions.

**Figure 4:**
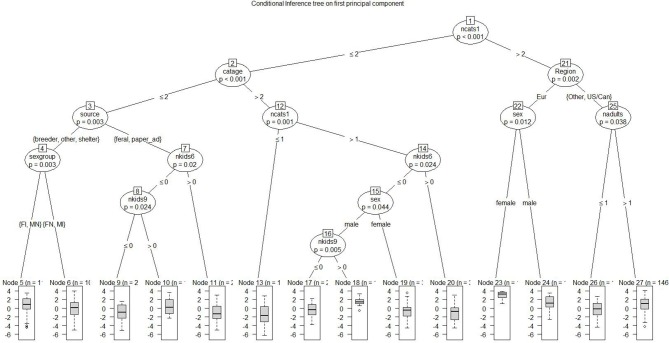
Conditional inference tree on first principal component: cat's positive interactions. each node represents a dichotomous split based on a demographic factor that distinguishes between lower-responding and higher-responding observations for the first principal component. As an example, node 1 shows that having more than 2 cats generally was favorable and associated with higher scores for cat's positive interactions with the child; this was especially true for cats in Europe (node 22) that were females (node 23). When having 2 cats or less that are 6 years of age or less, nodes 7 and 8 show that for cats living with no more than one other cat and no older than 6 years, and obtained as a feral or from an ad, having children age 9–12 at home was associated with the cat's positive interactions whereas having children age 6–8 was not. Statistical tests are noted at each node.

Additionally, three figures depicting conditional inference trees are presented, representing each of the three principal components, Prin 1, 2, and 3. This analysis searches recursively for predictors and threshold values (or dichotomous splits for categorical predictors) that result in a significant response difference, depending on whether the observation in question is above or below the threshold value. Figure 4 depicts Prin 1, *cat's positive interactions*, separating cats with high values of Prin1 from those with lower values; a high score is favorable. Solitary cats as a group had lower scores and younger cats had higher scores. The highest score at Node 23 reflected female cats in Europe living in families with at least 3 cats. Another high scorer, node 18 cats lived with one other cat, had no children 6–8, was a male cat, living with a child 9–12. As examples of cats with low scores, Node 13 represents solitary cats that are at least 6 years of age, and Node 9 includes cats living with no more than one other cat, that are no more than 6 years of age acquired as feral or from a newspaper ad, and lack any children 6–12 years old.

**Figure 5:**
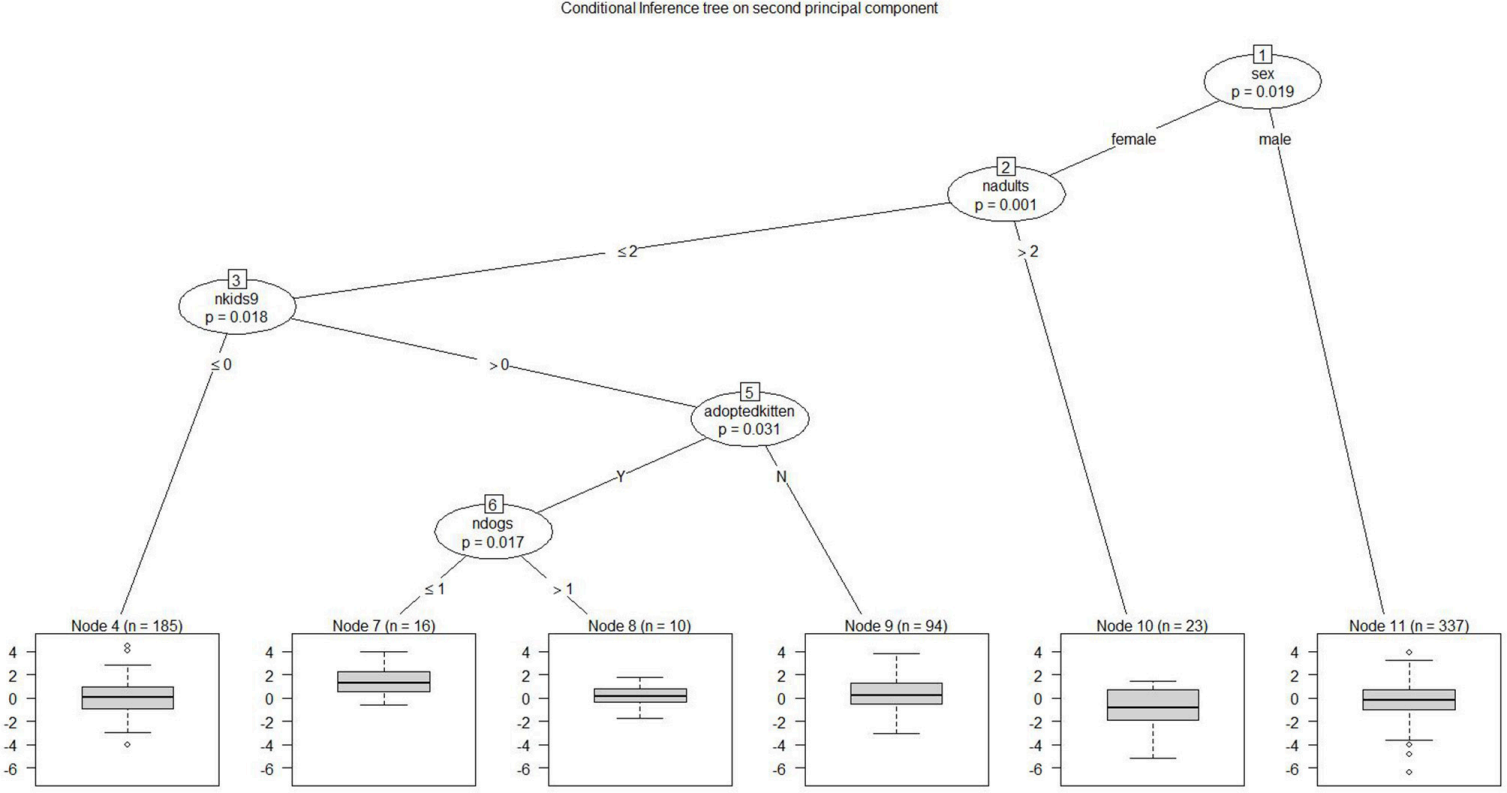
Conditional inference tree on second principal component: cat's fearfulness/aggression. each node represents a dichotomous split based on a demographic factor that distinguishes between lower-responding and higher-responding observations for the second principal component. Node 1 shows that female cats generally scored higher than male cats on fearfulness/aggression. Node 2 shows that having more than 2 adults in the home was associated with lower fearfulness/aggression. The most elevated scores for the factor of fearfulness/aggression were for female cats (node 1) living with up to 2 adults (node 2), living with at least one child 9–12 (node 3), adopted as a kitten (node 5), and living with a dog (node 6). Statistical tests are noted at each node.

Figure 5 depicts Prin 2, *fearfulness/aggression*, separating cats with high values of Prin 2 from those with lower values; a high score is unfavorable. The highest score at Node 7 included female cats living with 2 or fewer adults and a child 9–12 years old, adopted as a kitten, and living with one dog or less. The lowest score at Node 10 was for female cats living with 3 or more adults.

**Figure 6:**
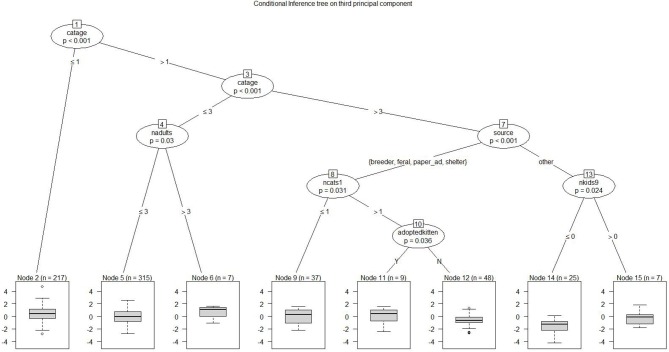
Conditional inference tree on third principal component: cat's playfulness and child's positive reaction. Each node represents a dichotomous split based on a demographic factor that distinguishes between lower-responding and higher-responding observations for the third principal component. For example, node 1 shows that female cats aged 1–3 years scored higher on playfulness with more child's positive reactions. At node 13, older cats acquired from other sources (such as a relative or friend) were more playful with children and had higher positive reactions from them if there were 9–12 year old children in the family. The lowest scores were for cats at least 10 years of age (nodes 1 and 3), adopted from a situation that was not breeder, feral, ad, or shelter (e.g., a neighbor or family member; node 7), living without a 9–12 year old (node 13). Statistical tests are noted at each node.

Figure 6 depicts Prin 3, *cat's playfulness and child's positive reaction*; a high score is favorable. The highest value at Node 6 represented a cat no more than middle-aged, living with at least 4 adults. The lowest score at Node 14 includes very old cats acquired from unusual sources with no children aged 9–12 at home, only younger children.

## Limitations of the research

The survey pertained to cat ownership, so participants necessarily were aware that the study was about cats. This knowledge presumably may have recruited participation of people who enjoy their cats and have good relationships with them. People whose cats have been aggressive may have relinquished those cats. The study was not a randomized survey representing all families with cats. In fact, in multi-cat households, respondents were asked to answer the survey with regard to the cat most interactive with the children.

## Discussion

This study focused on cats' affectionate and aggressive interactions with children. The web survey of the general public revealed that relationships of children with cats tend to be less consistently affectionate and are possibly more problematic than relationships cats have with adults. Many of the limitations in the relationships are from the cats' unwillingness to be affectionate.

Cats were generally more likely to be affectionate toward adults in the family than to children ages 3–5 years old. In considering the variables that could predict likelihood for a cat being very affectionate to children, the age of the cat, and fearfulness vs. friendliness toward visiting adults and/or children emerged as prominent factors that may result in conflicted relationships. Aggressiveness is obviously incompatible with being affectionate toward 3–5 year olds. Similar to our previous published study of cats with children who have autism (46), very few cats were reported as aggressive. The parents were asked to choose the cat that interacted with children the most, and answer questions regarding that cat. A strong majority of families had multiple cats, meaning that most children had the option to select a cat for interactions.

Many children would like their cats to be affectionate with them, but the cats may have less interest in a relationship than the children and may be unwilling to be held by a child. Early social habituation of kittens to children could predispose cats to be affectionate with young children. This would take advantage of the sensitive period in the early weeks of cats' lives when friendly, affectionate behavior can be elicited from cats (31–33).

Concerning the interesting result that cats in Europe were described more positively than those in other parts of the world, this may reflect different perceptions or expectations among respondents of what a cat's behavior is or should be, or it could involve unidentified differences based on living situations. With no direct observations of behaviors, it is not possible to know whether these differences reflect contrasts in the cats' behaviors.

Obtaining cats by a newspaper ad stood out as a risk factor, in the bi-plots. When obtaining a cat from a shelter or a breeder, the cat is receiving good care and efforts are made to locate a good home for the cat. Perhaps with a newspaper ad, there may be greater urgency to place the cat with less emphasis on the cat's care and welfare throughout the process.

Having at least two adults and multiple cats in the home was associated with more positive child-cat relationships, but as noted, the reason for this relationship is unclear. Old cats were the least satisfactory in child-cat relationships. Obtaining a kitten from a breeder or shelter seemed better than obtaining a cat that was feral, or from a newspaper ad, or other source. As previously highlighted (21, 22), the cat-human relationship is affected by both participants, and these results reveal that many variables can play a role in achieving a desirable relationship for a cat and child.

Cats living in Europe were rated as more interactive and less fearful than those living in the U.S./Canada. The higher rates of adoption of purebred cats and of kittens in Europe, rather than adopting older cats and acquiring cats from shelters as in the U.S./Canada, may help explain this difference.

In this survey of responding parents from the general public, presumably with mostly typically developing children and family cats, the cats varied in their levels of affection expressed to adults and children, with affection to adults being more common than to children, especially young children 3–5 years of age. Based on the responses from parents, children sought affectionate relationships with their cats and frequently enjoyed spending time with them. However, the desired level of a child's compatibility with the cat was often not fulfilled, in that some cats that are friendly and affectionate and provide a rewarding relationship to adults may offer much less to children in the family. This finding underscores the perspective that the cat's behavior is often the limiting factor in the interaction between a pet cat and a child, more than the child's level of interest. Also, these results suggest that with very young children 3–5 years of age, compatible relationships are more likely with younger cats. Risk factors for conflicted relationships include: the cat's age; fearful and aggressive behavior of the cat; and the cat's social context with other companion animals. Although isolated cats scored higher on aggression than those living with other animals, it is unclear whether the aggression led to a particular cat living with no other animals vs. the cat becoming aggressive due to isolation. Suggestions supported by the data in this study for enhancing compatible, affectionate relationships between children and cats are: (1) to assume that cats in the age range of 1 up to 6 years are more likely to be affectionate to very young children than older cats; and (2) not to assume that a cat that is fearless and affectionate toward adults will also be affectionate to young children. While there are behavioral differences among breeds of cats that would undoubtedly be important in predicting that a pet cat would be likely to be affectionate and non-aggressive with children (43), there were too few purebred cats in this study to address this issue.

## Ethics statement

The study was carried out in accordance with the recommendations of the University of California, Davis, Institutional Review Board as Protocols #201018447-1 and #284059-2. Participants responded anonymously to an on-line web survey and were informed on the survey of its voluntary nature, and that participation indicated their informed consent.

## Author contributions

LH, BH, and AT conceived and designed all phases of study, collected and analyzed data, and edited manuscript drafts. LH drafted and compiled manuscript. LL conceived and designed study. SH refined dataset entries for multi-variate statistical analyses by categorizing variables, especially for respondents' geographic status, cat acquisition as kittens, and numerical categories for statistical analyses. NW conceived and conducted extended statistical analyses, and drafted text for methods pertaining to figures that resulted, with specific edits. LH, BH, AT, NW, LL, and SH reviewed and edited interim and final draft manuscripts.

### Conflict of interest statement

The authors declare that the research was conducted in the absence of any commercial or financial relationships that could be construed as a potential conflict of interest.

## References

[1] Melson GF Studying children's attachment to their pets: a conceptual and methodological review. Anthrozoös (1990) 4:91–9. 10.2752/089279391787057297

[2] Turner D. A review of over three decades of research on cat-human and human-cat interactions and relationships. Behav Process. (2017) 141:297–304. 10.1016/j.beproc.2017.01.008 28119016

[3] Qureshi AI Memon MZ Vazquez G Suri MFK. Cat ownership and the risk of fatal cardiovascular diseases. Results from the second National Health and Nutrition Examination Study Mortality Follow-up Study. J Vasc Interv Neurol. (2009) 2:132–5. 22518240 PMC3317329

[4] Endenburg N van Lith HA. The influence of animals on the development of children. Vet J. (2011) 190:208–14. 10.1016/j.tvjl.2010.11.020 21195645

[5] Purewal R Christley R Kordas K Joinson C Meints K Gee N. Companion animals and child/adolescent development: a systematic review of the evidence. Int J Environ Res Public Health (2017) 14:234. 10.3390/ijerph14030234 28264460 PMC5369070

[6] Zasloff L Measuring attachment to companion animals: a dog is not a cat is not a bird. Appl Anim Behav Sci. (1996) 47:43–8.

[7] Brucke WF Cyno-psychoses. Children's thoughts, reactions, and feelings toward pet dogs. J Genetic Psych. (1903) 10:459–513. 10.1080/08919402.1903.10532729

[8] Guerney LF A survey of self-supports and social supports of self-care children. Element School Guid Counsel. (1991) 25:243–54.

[9] Endenburg N van Lith HA Kirpensteijn J Longitudinal study of Dutch children's attachment to companion animals. Soc Animals (2014) 22:390–414. 10.1163/15685306-12341344

[10] Borgi M Cirulli F Children's preferences for infantile features in dogs and cats. Hum Anim Interac Bull. (2013) 1:1–15. 10.1037/e634302013-002

[11] Levinson BM Pets and Human Development. Oxford: Charles C Thomas (1972).

[12] Black K Exploring Adolescent Loneliness and Companion Animal Attachment. Ph.D. Dissertation. Albuquerque: University of New Mexico (2009).

[13] Kidd AH Kidd RM Children's attitudes toward their pets. Psychol Rep. (1985) 57:15–31. 10.2466/pr0.1985.57.1.15 2377695

[14] Kidd AH Kidd RM Social and environmental influences on children's attitudes towards pets. Psychol Rep. (1990) 67:807–18. 10.2466/pr0.67.7.807-818 2377695

[15] Kidd AH Kidd RM. Children's drawings and attachment to pets. Psychol Rep. (1995) 77:235–41. 10.2466/pr0.1995.77.1.235 7501763

[16] Kidd AH Kidd RM Developmental factors in positive attitudes toward zoo animals. Psychol Rep. (1995) 76:71–81. 10.2466/pr0.1995.76.1.71

[17] Kidd AH Kidd RM Reactions of infants and toddlers to live and toy animals. Psychol Rep. (1987) 61:455–64. 10.2466/pr0.1987.61.2.455

[18] Bowlby J Attachment and Loss. Vol. 1 New York, NY: Basic Books (1969).

[19] Ainsworth M Blehar M Waters E Wall S Patterns of Attachment: Observations in the Strange Situation and at Home. Hillsdale, MI: Erlbaum (1978).

[20] Pika A Wilkinson R Kendrick K Vernes SC. Taking turns: bridging the gap between human and animal communication. Proc R Soc B Biol Sci. (2018) 285:20180598. 10.1098/rspb.2018.0598 29875303 PMC6015850

[21] Wedl M Bauer B Grace D Grabmayer C Spielauer E Day J Kotrschal K 2011. Factors influencing the temporal patterns of dyadic behaviours and interactions between domestic cats and their owners. Behav Processes (2011) 86:58–67. 10.1016/j.beproc.2010.09.001 20837114

[22] Kotrschal K Day J McCune S Wedl M Human and cat personalities: building the bond from both sides. In: Turner DC Bateson PG editors. The Domestic Cat. 3rd ed Cambridge: Cambridge University Press (2014). p. 113–28.

[23] Triebenbacher SL. Pets as transitional objects: their role in children's emotional development. Psychol Rep. (1998) 82:191–200. 10.2466/pr0.82.1.191-200 9520553

[24] Kosonen M Siblings as providers of support and care during middle childhood: children's perceptions. Child Soc. (1996) 10:267–79. 10.1111/j.1099-0860.1996.tb00595.x

[25] Bryant BK. The neighborhood walk: sources of support in middle childhood. Monographs Soc Res Child Dev. (1985) 50:122. 10.2307/3333847 4088288

[26] Melson GF Peet S Sparks C Children's attachment to their pets: links to socio-emotional development. Child Environ Q. (1991) 8:55–65.

[27] Daly B Morton LL An investigation of human-animal interactions and empathy as related to pet preference, ownership, attachment, and attitudes in children. Anthrozoös (2006) 19:113–27. 10.2752/089279306785593801

[28] Bennett PC Rutter NJ Woodhead JK Howell TJ. Assessment of domestic cat personality, as perceived by 416 owners, suggests six dimensions. Behav Processes (2017) 141:273–83. 10.1016/j.beproc.2017.02.020 28245980

[29] Finka LR The prediction of human-sociability in the domestic cat *Felis silverstris catus*. Ph.D. thesis. Lincoln: University of Lincoln (2015).

[30] Bennett V Gourkow N Mills DS. Facial correlates of emotional behaviour in the domestic cat (*Felis catus*). Behav Process. (2017) 141:342–50. 10.1016/j.beproc.2017.03.011 28341145

[31] Karsh EB 1983. The effects of early and late handling on the attachment of cats to people. In: Anderson RK Hart BL Hart LA editors. The Pet Connection. St. Paul, MN: Globe Press (1983). p. 207–15.

[32] Bradshaw J.W.S. The cat-human relationship. In: Bradshaw JWS editor. The Behaviour of the Domestic Cat. Wallingford: CAB International (1992). p. 163–76.

[33] Casey RA Bradshaw JWS The effects of additional socialization for kittens in a rescue centre on their behavior and suitability as a pet. Appl Anim Behav Sci. (2008) 114:196–205. 10.1016/j.applanim.2008.01.003

[34] Howell TJ Mornement K Bennett PC Pet cat management practices among a representative sample of owners in Victoria, Australia. J Vet Behav. (2016) 11:42–9. 10.1016/j.jveb.2015.10.006

[35] Fifield SJ Forsyth DK A pet for the children: factors related to family pet ownership. Anthrozoös (1999) 12:24–32. 10.2752/089279399787000426

[36] Murray JK Browne WJ Roberts MA Whitmarsh A Gruffydd-Jones TJ. Number and ownership profiles of cats and dogs in the UK. Vet Record (2010) 166:163–8. 10.1136/vr.b4712 20139379

[37] Westgarth C Heron J Ness AR Bundred P Gaskell RM Coyne KP. Family pet ownership during childhood: findings from a UK birth cohort and implications for public health research. Int J Environ Res Public Health (2010) 7:3704–29. 10.3390/ijerph7103704 21139856 PMC2996187

[38] Bjerke T Kaltenborn BP Ødegårdstuen TS Animal-related activities and appreciation of animals among children and adolescents. Anthrozoös (2001) 14:86–94. 10.2752/089279301786999535

[39] Marsa-Sambola F Williams J Muldoon J Lawrence A Connor M Roberts C Sociodemographics of pet ownership among adolescents in Great Britain: findings from the HBSC study in England, Scotland, and Wales. Anthrozoös (2016) 29:559–80. 10.1080/08927936.2016.1228756

[40] Adamelli S Marinelli L Normando S Bono G Owner and cat features influence the quality of life of the cat. Appl Anim Behav Sci. (2005) 94:89–98. 10.1016/j.applanim.2005.02.003

[41] Stammbach KB Turner DC Understanding the human—cat relationship: human social support or attachment. Anthrozoös (1999) 12:162–8. 10.2752/089279399787000237

[42] Arahori M Kuroshima H Hori Y Takagi S Chijiiwa H Fujita K. Owners' view of their pets' emotions, intellect, and mutual relationship: cats and dogs compared. Behav Process. (2017) 141:316–21. 10.1016/j.beproc.2017.02.007 28267573

[43] Hart BL Hart LA Your Ideal Cat: Insights into Breed and Gender Differences in Cat Behavior. West Lafayette, IN: Purdue University Press (2013). 147 p.

[44] Sueda K Hart BL Cliff KD Characterisation of plant eating in dogs. Appl Anim Behav Sci. (2008) 111:120–32. 10.1016/j.applanim.2007.05.018

[45] Gosling SD Vazire S Srivastava S John OP. Should we trust web-based studies? A comparative analysis of six preconceptions about internet questionnaires. Am Psychol. (2004) 59:93–104. 10.1037/0003-066X.59.2.93 14992636

[46] Hart LA Thigpen AP Willits NH Lyons LA Hertz-Picciotto I Hart BL. Affectionate interactions of cats with children having autism spectrum disorder. Front Vet Sci. (2018) 5:39. 10.3389/fvets.2018.00039 29594156 PMC5862067

